# Prospective, non-interventional study of ruxolitinib therapy in patients with polycythaemia vera – German real-world data (PaVe study)

**DOI:** 10.1007/s00277-026-06947-9

**Published:** 2026-05-12

**Authors:** Martin Griesshammer, Eyck von der Heyde, Rudolf Schlag, Burkhard Schmidt, Steffi Weniger, Michael Koenigsmann, Martina Stauch, Eva Gückel, Volker Baum, Rudolf Weide

**Affiliations:** 1 University Clinic for Hematology and Oncology, Johannes Wesling Clinic Minden, University Bochum, Minden, Germany; 2 Oncology at Raschplatz, Hannover, Germany; 3 Hematology-Oncology Specialist Practice, Würzburg, Germany; 4 Hematology and Oncology München-Pasing MVZ GmbH, München, Germany; 5 Collective Practice, Erfurt, Germany; 6 Oncology Outpatient Center, Hannover, Germany; 7 Outpatient Center for Hematology, Oncology and Coagulation, Kronach, Germany; 8 Novartis Pharma GmbH, Nürnberg, Germany; 9 InVo, Institute for Health Services Research Oncology GbR, Koblenz, Germany

**Keywords:** PaVe non-interventional study, Ruxolitinib, Polycythemia vera, Real-world evidence

## Abstract

Ruxolitinib, a JAK1/JAK2 inhibitor, has demonstrated efficacy in achieving hematocrit control, spleen volume reductions, and symptom improvement in patients with polycythaemia vera (PV) resistant to or intolerant of hydroxyurea in clinical trials. PaVe is a prospective, noninterventional study of patients with PV who received ruxolitinib in a real-world setting in Germany. Observational parameters included effectiveness, safety, patient satisfaction and quality of life. Patients with and without prior treatment with ruxolitinib were included in this study. Additionally, patients were stratified by no/high risk. The study was primarily analyzed using descriptive methods. Four hundred and thirty-three patients with PV were included in this study, of which 62% were pretreated with a JAK-inhibitor and 38% were not. Mean decreases from Baseline in hematocrit levels/leukocyte counts at the last assessment were more pronounced in naïve patients (-6.2% versus − 1.6%/-4.5 × 10^3^/µl versus − 1.4 × 10^3^/µl) and high-risk patients (-6.3% versus − 5.3%/-4.8 × 10^3^/µl versus − 2.6 × 10^3^/µl). Mean decreases from Baseline in thrombocyte counts at the last assessment were more pronounced in naïve patients (-80.5 × 10^3^/µl versus − 42.6 × 10^3^/µl) and no-risk patients (-108.0 × 10^3^/µl versus − 77.1 × 10^3^/µl). There was almost no difference in the total number of thromboembolic events in JAK inhibitor naïve versus JAK inhibitor pretreated patients (3.1%, n = 5 versus 3.8%, n = 10). No new safety concerns were observed with ruxolitinib. Findings from this study in a real-world setting show that ruxolitinib is an effective and safe second-line treatment in PV patients.

## Background

 Polycythaemia vera (PV) is one of the three classic BCR-ABL negative myeloproliferative neoplasms (MPN) [1], along with myelofibrosis (MF) and essential thrombocythemia (ET). Worldwide, one to three people per 100,000 develop PV [2] each year, making it the most common MPN [3].

PV is a clonal disease of hematopoietic stem cells that results in increased cell growth of one or more blood cell lines. An increase in erythrocyte mass is the most typical clinical manifestation; increases in leukocyte and platelet counts also occur, although less frequently [4]. At the beginning or during the course of the disease severe symptoms occur (e.g. pruritus, fatigue, splenomegaly associated problems), which strongly affect the patients’ quality of life [5]. PV patients have a shorter life expectancy compared to the general population: the median survival time after diagnosis is 12 to 19 years [6–8]. The most common causes of death are cardio- and cerebrovascular complications and transformation to post-PV MF or acute myeloid leukemia (AML) [9].

The achievement of a lower hematocrit (HCT) with a target HCT < 45% plays a prominent role in the prevention of thromboembolic complications, as demonstrated in a randomized, prospective, phase III study (CYTO-PV). Patients whose HCT was adjusted to a target value below 45% had a fourfold lower rate of fatal thromboembolic complications compared to patients whose HCT was adjusted between 45% and 50% [10]. Accordingly, standard PV therapy includes the use of aspirin and phlebotomy. Patients who have an increased risk of thrombosis and/or do not tolerate phlebotomies, or have disease progression, receive additional cytoreductive treatment with hydroxyurea (HU) or interferon being the two most frequently used first line cytoreductive treatments [11–13].

However, a certain proportion of patients develop HU resistance or intolerance (11% and 13%, respectively), which complicates effective treatment of the disease and results in an increased death rate [8]. Demuynck et al. reported an even higher rate of HU resistant/intolerant patients when comparing the original European Leukemia Net criteria versus the modified European Leukemia Net criteria (20.7% versus 39.6%) [14]. Treatment with phlebotomies is also not always without problems, as it is associated with poor compliance, iron deficiency and an increased risk of thrombosis [15]. Thus, there is a high medical need for other therapeutic modalities.

Jakavi^®^ (ruxolitinib) is an oral Janus kinase (JAK) inhibitor that selectively inhibits JAK1 and JAK2. It is approved by the European Commission for the treatment of MF and for the treatment of adults with PV who are intolerant or resistant to HU. The approval was based on data from a randomized, multicenter Phase III study (RESPONSE) in which 222 PV patients with splenomegaly were randomized to receive either ruxolitinib or best available therapy (BAT) and treated for 80 weeks [16]. Together, the two phase III trials RESPONSE [17] and RESPONSE-2 [18, 19] showed that ruxolitinib was superior to best available therapy in controlling HCT, improving PV-related symptoms, and in reducing spleen volume in patients with PV resistant to or intolerant of HU.

The aim of this NIS was to obtain data from standard clinical routine on ruxolitinib therapy of PV in a broad patient population.

## Methods

### Patients and study oversight

This NIS was planned to be conducted in a multicenter setting at approximately 125 hematology/oncology practices and hospitals in Germany with a target population of 500 PV patients. All patients were aged ≥ 18 years, diagnosed according to World Health Organization (WHO, 2016) and were resistant/intolerant to HU. All patients had provided written informed consent. The study was approved by the ethics committee of the “Ärztekammer Westfalen-Lippe und der Westfälischen Wilhelms Universität” (reference number: 2015-195-f-S). The patients were divided into 2 groups: patients without prior treatment with ruxolitinib or other JAK inhibitor (about 250) and patients pretreated with ruxolitinib or other JAK inhibitor (about 250). Prospective data on outcomes, treatment, symptom burden, and safety were obtained at an interval according to routine care, the clinical symptoms of the particular patient, and the requirements of the current professional information, e.g.: 1, 2, 3, 6, 9, 12, 18, 24, 30 and 36 months after the initial examination and start of treatment with ruxolitinib. The study was funded by Novartis Pharma.

### Objectives of the study

In order to assess the immediate treatment effect, patients without prior treatment with ruxolitinib (naïve patients) were included in this NIS; at the same time, pretreated patients were documented in order to better investigate the long-term effectiveness of ruxolitinib therapy.

The following main objectives were of interest: Investigation of the effectiveness of ruxolitinib in standard clinical practice based on the HCT, leukocyte, and thrombocyte value, spleen size, as well as the symptom burden. Furthermore, an extended investigation of the tolerability and safety of ruxolitinib in standard clinical practice based on the total number of adverse events (AEs) and serious adverse events (SAEs, with and without causal relationship to ruxolitinib) was subject of the study. Additionally, the effectiveness of ruxolitinib between different risk groups was assessed (age > 60 years, prior thromboembolic event).

### Statistics

The analysis set (AS) comprised all patients who fulfilled all inclusion criteria (diagnosis of PV, treatment with ruxolitinib indicated, written informed consent), received at least one dose of ruxolitinib during the study and performed the Baseline visit.

The NIS was primarily analyzed using descriptive methods. Where confidence intervals (CI) or p-values are given, these are also purely descriptive. Due to the exploratory nature of the analysis, no alpha adjustment was made for multiple comparisons.

For HCT, erythrocyte, thrombocyte and leukocyte values, the course and the difference to Baseline were analyzed by visit and history of JAK inhibitors.

High risk was defined by older than or equal to 60 years and/or history of thromboembolic events.

A modified Myeloproliferative Neoplasm Symptom Assessment Form (MPN-10 SAF) was used to calculate symptom severity and quality of life. Total symptom score (TSS, score ranges from 1 to 100) was calculated based on 10 individual symptoms.

For AEs, incidences based on the included patient population and incidence densities (number of events/sum of person-times in years) were reported according to Medical Dictionary for Regulatory Activities (MedDRA) System Organ Class (SOC) and Preferred Term (PT) for AEs, serious adverse events (SAEs), adverse drug reactions (ADRs), and serious adverse drug reactions (SADRs). All patients who had received at least one dose of ruxolitinib and for whom at least one follow-up documentation was available were included in the analysis.

## Results

### Baseline characteristics

Starting in August 2015, 433 patients (median age: 72 years) were included in the analysis set (AS), of which 62% (*n* = 268) were pretreated with a JAK inhibitor and 38% (*n* = 165) were JAK inhibitor naïve (Table 1). Patients previously treated with a JAK inhibitor were slightly younger, were more fully active, had a lower median HCT value, a lower symptom burden, and had undergone less phlebotomies at Baseline. Almost 90% of the patients were at high risk (e.g., being ≥ 60 years old and/or having a history of thromboembolic events).

**Table 1:** General characteristics and risk status of the 433 PV patients at Baseline

Variable	JAK inhibitor naive (*N* = 165)	JAK inhibitor pretreated (*N* = 268)	Total (*N* = 433)
Age (years)			
Median (range)	74.0 (26.0 to 91.0)	71.0 (33.0 to 90.0)	72.0 (26.0 to 91.0)
**Sex**,** n (%)**			
Male	89 (53.9)	147 (54.9)	236 (54.5)
Female	76 (46.1)	121 (45.1)	197 (45.5)
**Time since diagnosis**,** months**			
Median (range)	89.2 (0.2 to 384.4)	85.2 (0.3 to 361.5)	86.0 (0.2 to 384.4)
**Duration of pretreatment**,** months**			
Median (range)	-	4.8 (0.2 to 72.3)	-
**ECOG performance status**,** n(%)**			
0	60 (45.5)	108 (56.0)	168 (51.7)
1	65 (49.2)	71 (36.8)	136 (41.8)
2	7 (5.3)	11 (5.7)	18 (5.5)
3	-	3 (1.6)	3 (0.9)
**HCT**			
Median (range)	44.3 (24.0 to 62.7)	39.0 (23.9 to 57.2)	40.7 (23.9 to 62.7)
**Phlebotomies since diagnosis**			
Median (range)	13.0 (0.0 to 103.0)	10.0 (0.0 to 250.0)	10.0 (0.0 to 250.0)
**Age (category)**			
Age ˂ 60 year	27 (16.4)	47 (17.5)	74 (17.1)
Age ≥ 60 years	138 (83.6)	221 (82.5)	359 (82.9)
**Thromboembolic event**,** n(%)**			
Absence at Baseline	114 (69.1)	180 (67.2)	294 (67.9)
Presence at Baseline	51 (30.9)	88 (32.8)	139 (32.1)
**Patients at high risk**^**a**^, **n (%)**			
No high risk	20 (12.1)	29 (10.8)	49 (11.3)
With high risk	145 (87.9)	239 (89.2)	384 (88.7)
**Modified MPN-10 SAF TSS**			
Median (range)	28.0 (8.0 to 83.0)	24.0 (1.0 to 65.0)	25.0 (1.0 to 83.0)

*ECOG* Eastern Cooperative Oncology Group, *HCT* hematocrit, *JAK* Janus Kinase, *MPN SAF* Myeloproliferative Neoplasm Symptom Assessment Form, *SD* standard deviation, *TSS* total symptom score

The ECOG performance status ranges from 0 to 5 where 0 = fully active, able to carry on all pre-disease performance without restriction, 1 = restricted in physically strenuous activity but ambulatory and able to carry out work of a light or sedentary nature, e.g., light house work, office work, 2 = ambulatory and capable of all selfcare but unable to carry out any work activities; up and about more than 50% of waking hours, 3 = capable of only limited selfcare; confined to bed or chair more than 50% of waking hours, 4 = completely disabled; cannot carry on any selfcare; totally confined to bed or chair, 5 = dead

Modified MPN-10 SAF: MPN-SAF TSS has a possible range of 1 to 100, with 100 representing the highest level of symptom severity

^a^ High risk was defined by older or equal than 60 years and/or history of thromboembolic events

### Patient disposition and treatment during the study

In total, median duration of treatment with ruxolitinib was 34.5 months, ranging from 0.03 (corresponding to 1 day) to 45.6 months. The median daily dose of ruxolitinib was 20.0 mg, ranging from 3.9 to 47.9 mg.

Overall, 65.6% (*n* = 284) of the patients completed the study and 34.4% (*n* = 149) did not. The three most frequent reasons for premature discontinuation were “death” (8.1%), “adverse event” (7.9%), and “lost to follow up” (7.6%, Table 2).

**Table 2:** Patient disposition

Event	JAK inhibitor naive (*N* = 165) *n* (%)	JAK inhibitor pretreated (*N* = 268) *n* (%)	Total (*N* = 433) *n* (%)
Study completed			
Yes	109 (66.1)	175 (65.3)	284 (65.6)
No	56 (33.9)	93 (34.7)	149 (34.4)
**Reason for premature discontinuation** ^**a**^			
Death	8 (4.8)	27 (10.1)	35 (8.1)
Adverse event	14 (8.5)	20 (7.5)	34 (7.9)
Lost-to-follow-up	11 (6.7)	22 (8.2)	33 (7.6)
Other	13 (7.9)	16 (6.0)	29 (6.7)
Deterioration of general health	6 (3.6)	6 (2.2)	12 (2.8)
Poor compliance	6 (3.6)	4 (1.5)	10 (2.3)
Progression of primary disease	1 (0.6)	9 (3.4)	10 (2.3)
No therapy response	6 (3.6)	1 (0.4)	7 (1.6)
Infections	-	1 (0.4)	1 (0.2)

*AK* Janus Kinase

^a^ Multiple answers possible

### Effectiveness

**Fig. 1:**
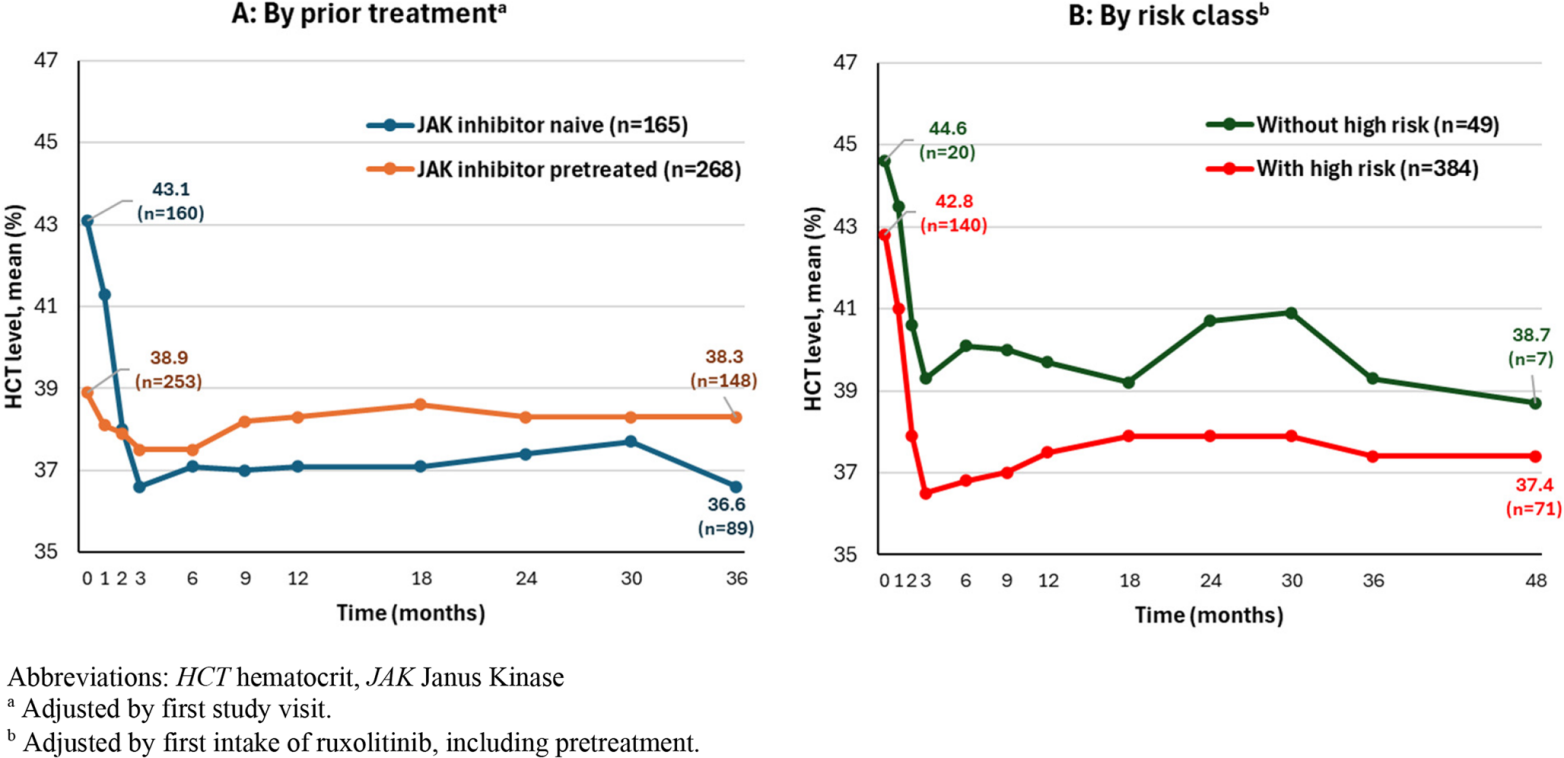
HCT levels over time

**Fig. 2:**
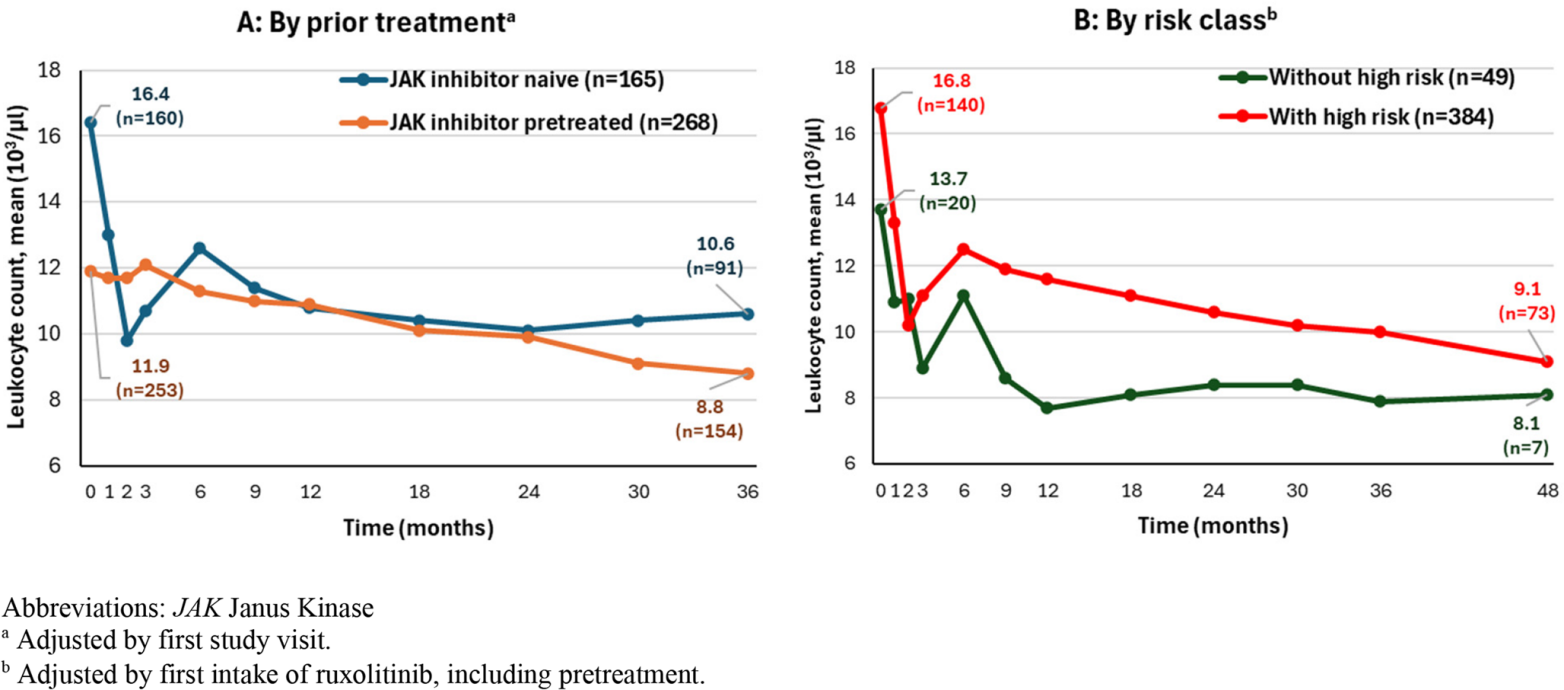
Leukocyte counts over time

**Fig. 3:**
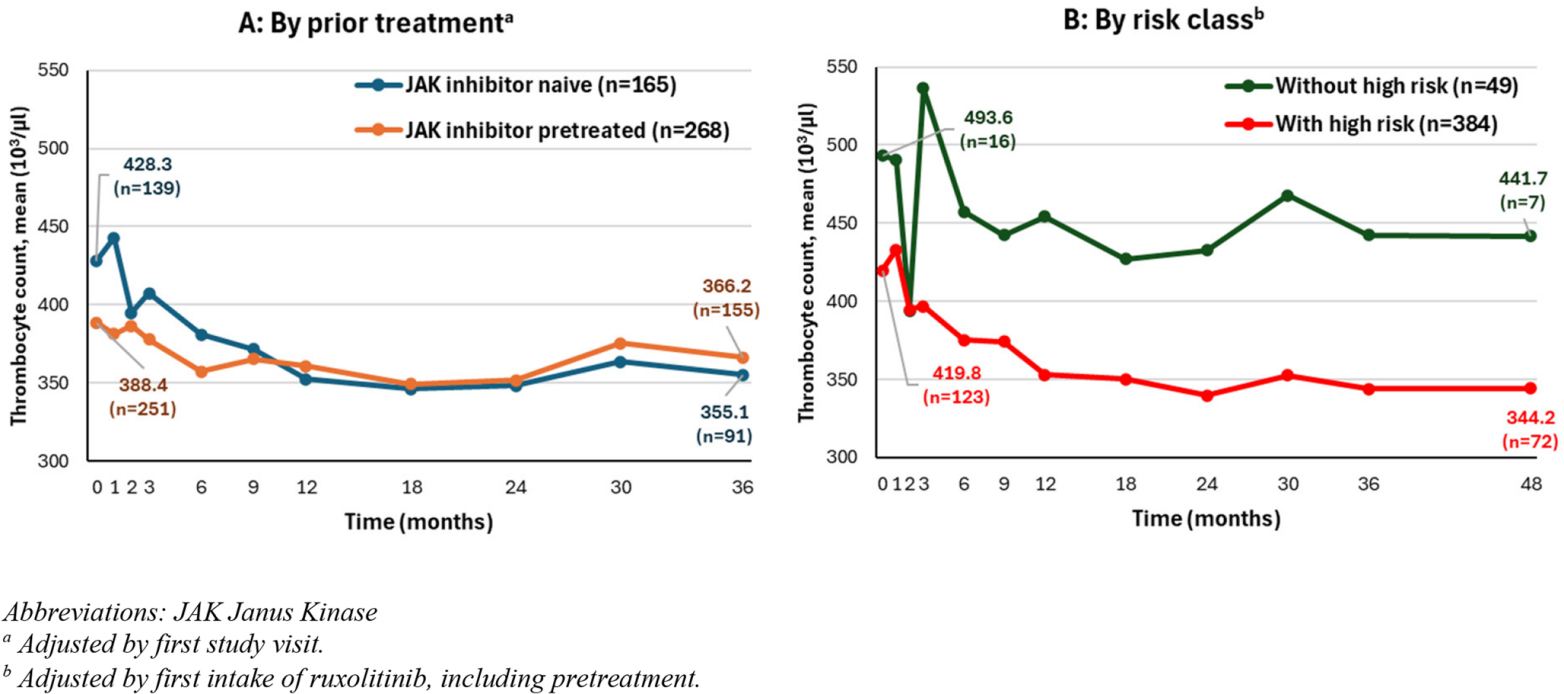
Thrombocyte counts over time

Figures 1, 2 and 3, and 5 show the course of specific parameters from Baseline to Month 36/48, once stratified according to JAK inhibitor pre-treatment (A, adjusted by first visit in the study) and once according to risk class (B, adjusted by the first application of ruxolitinib).

#### HCT levels

JAK inhibitor naïve patients (43.1% ± 6.4%, interquartile range [IQR] 39.2 to 47.4%) started from a higher mean (± SD) HCT level compared to JAK inhibitor pretreated patients (38.9% ± 6.0%, IQR 35.0 to 43.0%). In JAK inhibitor naïve patients, the mean HCT values rapidly decreased within the first 3 months to a level comparable to JAK inhibitor pretreated patients. From Month 3 on, HCT levels maintained and had a similar course for JAK inhibitor naïve and JAK inhibitor pretreated patients. At Month 36, mean HCT value was 36.6% ± 4.8% for JAK inhibitor naïve patients (IQR 34.2 to 39.2%) and 38.3% ± 5.1% for JAK inhibitor pretreated patients (IQR 34.8 to 42.0%, Fig. 1A).

In both risk groups mean HCT values rapidly decreased from Baseline (*without high risk*: 44.6% ± 7.0%, IQR 42.0 to 49.2%; *with high risk*: 42.8% ± 6.3%, IQR 38.9 to 47.2%) to Month 3 (*without high risk*: 39.3% ± 5.9%, *n* = 31, IQR 34.5 to 44.0%; *with high risk*: 36.5% ± 5.7%, *n* = 215, IQR 32.3 to 41.0%). Patients with high risk had lower mean HCT values during the study compared to patients without high risk (Fig. 1B).

#### Leukocyte counts

Here too, JAK inhibitor naïve patients (16.4 ± 11.8 × 10^3^/µl, IQR 7.6 to 20.7 × 10^3^/µl) started from a higher mean (± SD) leukocyte count compared to JAK inhibitor pretreated patients (11.9 ± 9.7 × 10^3^/µl, IQR 6.7 to 12.9 × 10^3^/µl). A sustained, rapid reduction of mean leukocyte values in JAK inhibitor naïve patients to a level comparable to JAK inhibitor pretreated patients was seen within the first 2 months. From Month 2 on, mean leukocyte values had a similar course for JAK inhibitor naïve and JAK inhibitor pretreated patients. At Month 36, mean leukocyte value was 10.6 ± 9.1 × 10^3^/µl for JAK-inhibitor naïve patients (IQR 5.6 to 12.4 × 10^3^/µl) and 8.8 ± 5.1 × 10^3^/µl for JAK inhibitor pretreated patients (IQR 5.6 to 9.8 × 10^3^/µl, Fig. 2 A).

In both risk groups mean leukocyte values dropped quickly from Baseline (*without high risk*: 13.7 ± 11.6 × 10^3^/µl, IQR 7.0 to 13.3 × 10^3^/µl; *with high risk*: 16.8 ± 11.8 × 10^3^/µl, 7.7 to 21.3 × 10^3^/µl) to Month 2 (*without high risk*: 11.0 ± 13.4 × 10^3^/µl, *n* = 20, IQR 6.0 to 10.5 × 10^3^/µl; *with high risk*: 10.2 ± 7.8 × 10^3^/µl, *n* = 149, IQR 5.6 to 12.0 × 10^3^/µl). Patients without high risk had lower leukocyte values during the study compared to patients with high risk (Fig. 2B).

#### Thrombocyte counts

JAK-inhibitor naïve patients (428.3 ± 213.6 × 10^3^/µl, IQR 244.0 to 569.0 × 10^3^/µl) started from a higher mean (± SD) thrombocyte count compared to JAK inhibitor pretreated patients (388.4 ± 202.3 × 10^3^/µl, IQR 249.0 to 497.0 × 10^3^/µl). From Month 3 on, thrombocyte values had almost the same course for JAK inhibitor naïve and JAK inhibitor pretreated patients. At Month 36, mean thrombocyte value was 355.1 ± 167.2 × 10^3^/µl for JAK inhibitor naïve patients (IQR 231 to 482.0 × 10^3^/µl) and 366.2 ± 160.6 × 10^3^/µl for JAK inhibitor pretreated patients (IQR 262.0 to 443.0 × 10^3^/µl, Fig. 3 A).

Mean thrombocyte values were lower in high-risk than low risk patients (Fig. 3B). Here, the number of patients without high risk and available thrombocyte values was low (*n* = 16), not allowing a direct comparison.

#### Spleen length over time

A sonography was performed in 168 out of 433 patients (38.8%) at Baseline. Spleen length values were available for 150 patients at Baseline, resulting in a mean spleen length of 15.8 ± 4.3 cm, ranging from 6.7 cm to 30.0 cm (median 15.0 cm).

A last post-Baseline visit sonography value was available for 228 patients. Here, mean spleen length was 13.7 ± 3.8 cm (median 12.9 cm, ranging from 7.0 to 30.0 cm). The mean difference to Baseline could be calculated for 110 patients and was − 1.88 ± 3.1 cm (median − 1.9 cm, ranging from − 10.8 to 7.2 cm).

**Fig. 4:**
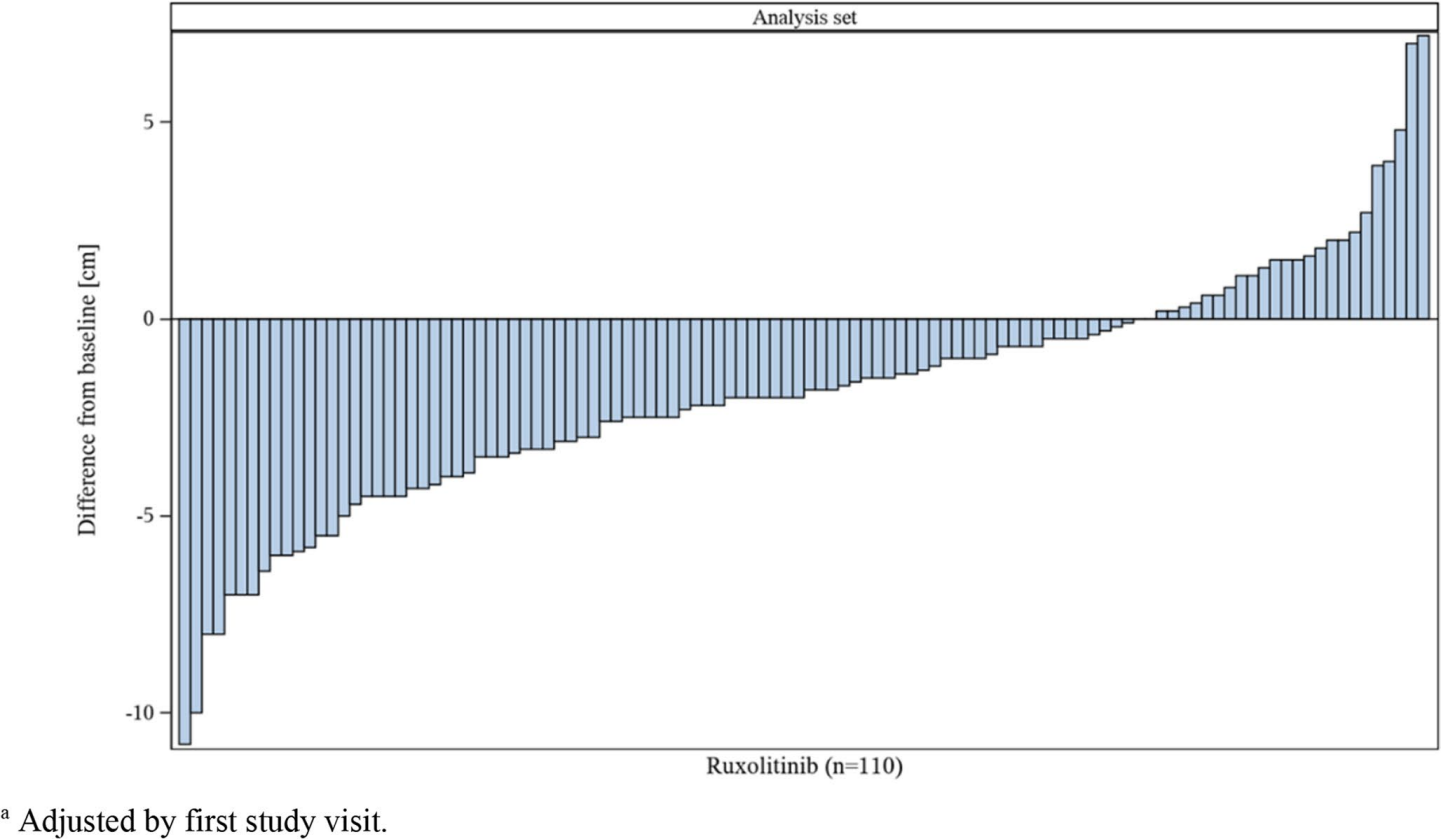
Waterfall Plot for change in spleen length over time^a^

Figure 4 displays a Waterfall Plot for the difference in spleen length from Baseline to the last post-Baseline visit regardless of JAK inhibitor pretreatment. JAK inhibitor led to a reduction in spleen length for the majority of patients, shown on the left side of the graph as negative difference to Baseline.

### Phlebotomies

In total, for 421 patients (162 JAK-inhibitor naïve and 259 JAK-inhibitor pretreated patients) data was available regarding the performance of a phlebotomy within 30 days prior to Baseline. More than 91% of the JAK-inhibitor pretreated patients had no phlebotomy within the last month, compared to 74% in JAK-inhibitor naïve patients. Median time since last phlebotomy prior to Baseline was 4.1 months in JAK-inhibitor naïve patients and 13.5 months in JAK-inhibitor pretreated patients.

Irrespective of JAK-inhibitor pretreatment, more than 80% of the patients were phlebotomy-free per study year. The mean total number of phlebotomies per year was 0.3 ± 1.0 (median: 0.0, ranging from 0.0 to 11.0) for JAK-inhibitor naïve patients and 0.2 ± 1.1 (median: 0.0, ranging from 0.0 to 13.4) for JAK-inhibitor pretreated patients.

Regardless of risk classification, more than 80% of the patients did not need a phlebotomy per study year. The mean total number of phlebotomies per year was 0.5 ± 1.7 (median: 0.0, ranging from 0.0 to 11.0) for patients without high risk and 0.2 ± 0.9 (median: 0.0, ranging from 0.0 to 13.4) for patients with high risk.

### Thromboembolic events

The number of thromboembolic events per year was low regardless of JAK inhibitor pretreatment and ranged from 0 to a maximum of 2.1 events. In total, 3.5% (*n* = 15) of the patients had at least one thromboembolic event (Table 3).

**Table 3:** Number of thromboembolic events

Variable	JAK inhibitor naive (*N* = 165)	JAK inhibitor pretreated (*N* = 268)	Total (*N* = 433)
Thromboembolic events per 100 patient-years			
n	162	265	427
Mean ± SD	2.5 ± 18.7	1.9 ± 11.6	2.1 ± 14.7
Median (range)	0.0 (0.0 to 210.0)	0.0 (0.0 to 150.0)	0.0 (0.0 to 210.0)
**Total number of thromboembolic events**,** n (%)**			
0	157 (96.9)	255 (96.2)	412 (96.5)
≥ 1	5 (3.1)	10 (3.8)	15 (3.5)
Missing values	3	3	6

*JAK* Janus Kinase, *SD* standard deviation

The thromboembolic events could be specified more precisely via tick boxes in the documentation, with the following options: stroke, acute coronary syndrome, transient ischaemic attack, pulmonary embolism, thrombosis, deep vein thrombosis, peripheral arterial thrombosis, and other, whereby the latter should be documented as free text. Six thromboembolic events were assessed with severe intensity, 1 event in JAK inhibitor naïve and 5 events in JAK inhibitor pretreated patients: pulmonary embolism (*n* = 3), acute coronary syndrome (*n* = 2), other (erysipelas lower leg on both sides was inconsistently documented as free text, *n* = 1; Table 4).

**Table 4:** Type and intensity of thromboembolic events

Type Intensity	JAK inhibitor naive (*N* = 165) *n* (%)	JAK inhibitor pretreated (*N* = 268) *n* (%)	Total (*N* = 433) *n* (%)
Any thromboembolic event	5 (3.0)	10 (3.7)	15 (3.5)
Stroke	-	1 (0.4)	1 (0.2)
Moderate	-	1 (0.4)	1 (0.2)
**Acute coronary syndrome**	**-**	**2 (0.7)**	**2 (0.5)**
Severe	-	2 (0.7)	2 (0.5)
**Transient ischaemic attack**	**-**	**1 (0.4)**	**1 (0.2)**
Mild	-	1 (0.4)	1 (0.2)
**Pulmonary embolism**	**2 (1.2)**	**3 (1.1)**	**5 (1.2)**
Moderate	2 (1.2)	-	2 (0.5)
Severe	-	3 (1.1)	3 (0.7)
**Thrombosis**	**-**	**1 (0.4)**	**1 (0.2)**
Mild	-	1 (0.4)	1 (0.2)
**Deep vein thrombosis**	**-**	**1 (0.4)**	**1 (0.2)**
Moderate	-	1 (0.4)	1 (0.2)
**Peripheral arterial thrombosis**	**-**	**-**	**-**
**Other**	**3 (1.8)**	**1 (0.4)**	**4 (0.9)**
Mild	1 (0.6)	-	1 (0.2)
Moderate	2 (1.2)	1 (0.4)	3 (0.7)
Severe	1 (0.6)	-	1 (0.2)

*JAK* Janus Kinase

### Symptom burden

**Fig. 5:**
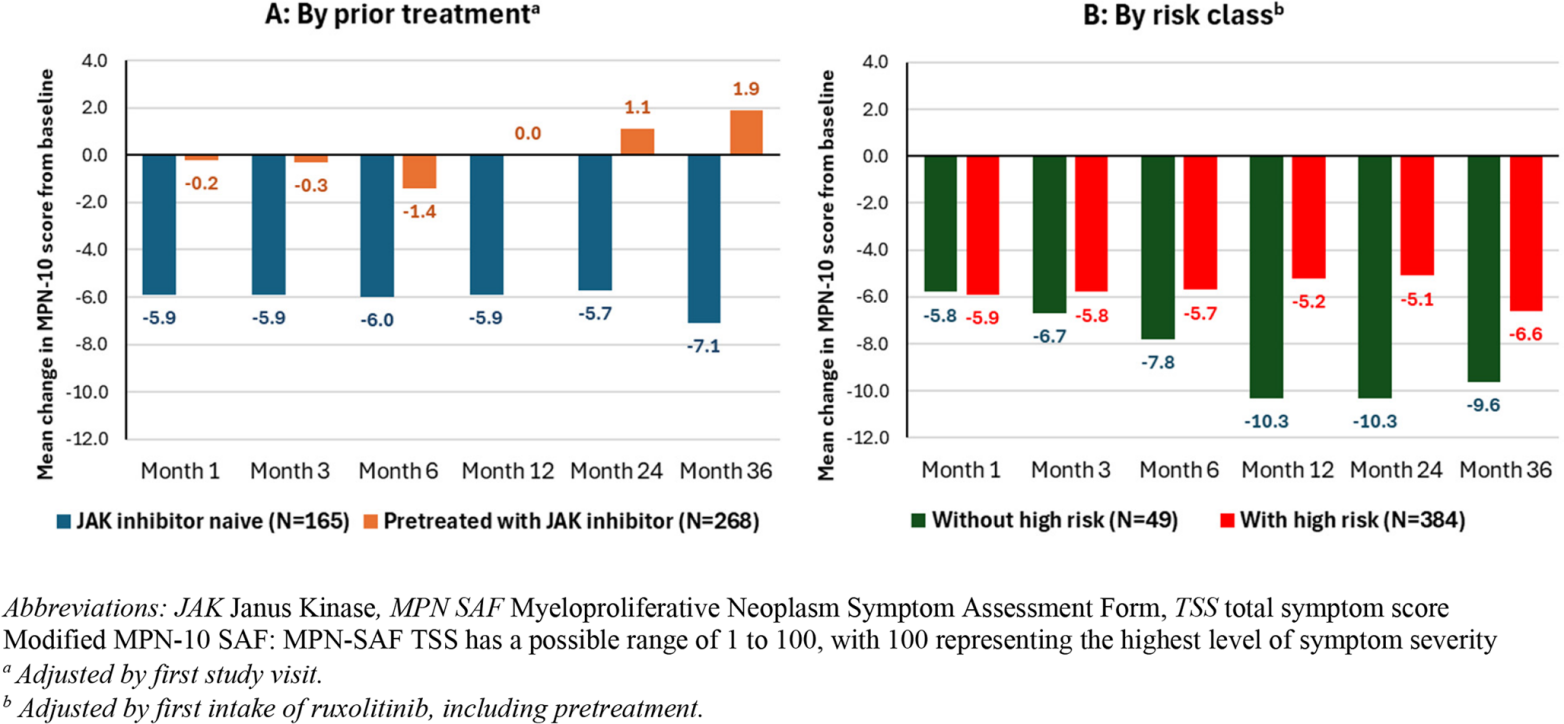
Mean difference to Baseline in modified MPN-10 SAF TSS over time

Mean decrease of TSS (symptom burden as assessed by a modified MPN-10 questionnaire) was consistent from Month 1 (−5.9 ± 11.6, n = 111) to Month 36 (−7.1 ± 12.5, n = 43) in JAK-inhibitor naïve patients. JAK-inhibitor pretreated patients maintained their mean MPN-10 scores from Month 1 (−0.2 ± 11.2, n = 129) to Month 36 (1.9 ± 11.9, n = 84, Fig. 5A). Looking at the risk status, patients without high risk had higher mean decreases in TSS from − 5.8 ± 10.3 (n = 14) at Month 1 to −9.6 ± 14.6 (n = 7) at Month 36, compared to patient with high risk, who remained stable from Month 1 (−5.9 ± 11.9, n = 97) to Month 36 (−6.6 ± 12.2, n = 36, Fig. 5B).

### Exposure and safety

In total, median duration of treatment with ruxolitinib was 34.5 months. There was almost no difference in the treatment duration between JAK-inhibitor naïve and JAK-inhibitor pretreated patients (34.0 versus 34.5 months). Median dose of ruxolitinib per day was 20 mg/day irrespective of pretreatment with ruxolitinib.

Overall, the majority of patients (82.4%, *n* = 357) had no interruption of ruxolitinib therapy. 12.7% (*n* = 55) of the patients had 1 interruption of ruxolitinib therapy, mostly due to an AE (60.7%). Median duration for all patients of the interruption of ruxolitinib therapy was 24 days ranging from 1 to 206 days. The results of the evaluation of the interruptions of ruxolitinib therapy were comparable in the subgroups (no pretreatment/pretreatment with ruxolitinib).

Out of 165 JAK inhibitor naïve patients, 147 patients (89.1%) experienced at least 1 AE. Out of these, 59 patients (35.8%) experienced 107 SAEs, in 12 patients (7.3%), these were drug related (15°SADRs).

Out of 268 JAK inhibitor pretreated patients, 229 patients (85.4%) experienced at least 1 AE. Out of these, 101 patients (37.7%) experienced 238 SAEs, in 25 patients (9.3%), these were drug related (38SADRs; Table 5).

**Table 5:** Summary of adverse events

Variable	JAK-inhibitor naive (*N* = 165)	JAK-inhibitor pretreated (*N* = 268)	Total (*N* = 433)
Number of patients with, *n* (%)			
** AE**	147 (89.1)	229 (85.4)	376 (86.8)
** nsAE**	140 (84.8)	212 (79.1)	352 (81.3)
nsADR	88 (53.3)	133 (49.6)	221 (51.0)
nsAEnr	119 (72.1)	186 (69.4)	305 (70.4)
** SAE**	59 (35.8)	101 (37.7)	160 (37.0)
SADR	12 (7.3)	25 (9.3)	37 (8.5)
SAEnr	54 (32.7)	95 (35.4)	149 (34.4)
**Number of events**			
** AE**	798	1172	1970
** nsAE**	691	934	1625
nsADR	209	264	473
nsAEnr	482	670	1152
** SAE**	107	238	345
SADR	15	38	53
SAEnr	92	200	292

*ADR* adverse drug reaction, *AE* adverse event, *JAK* Janus Kinase, *nr* not related, *ns* non-serious, *SADR* serious adverse drug reaction, *SAE* serious adverse event

*AEs *without information on relation to study drug or seriousness were considered as related or serious, respectively

In total, anemia (25.2%, n = 109) was the most common hematologic AE; fatigue (11.3%, n = 49), and dizziness (10.2%, n = 44), were the most common non-hematologic AEs. Anemia of Grade 3 or higher occurred in 11 patients (2.5%) out of 433 patients at risk, fatigue and dizziness in 3 patients each (0.7% each).

Serious AEs with a causal relationship to ruxolitinib (SADR) were rare and were reported in 4 patients (0.9%) with decreased hemoglobin, 3 patients (0.7%) with increased weight, and 1 patient each (0.2%) with anemia, dizziness, thrombocytopenia, and herpes zoster (Table 6, showing AEs if present in ≥ 5% of the patients).

**Table 6:** Most common AEs by patient (if present in ≥ 5% of the patients)

AS (433 patients) Number of patients with *n*(%)	nsAEnr	nsADR	nsAE	SAEnr	SADR	SAE	AE
Anaemia	43 (9.9)	72 (16.6)	107 (24.7)	5 (1.2)	1 (0.2)	6 (1.4)	109 (25.2)
Fatigue	30 (6.9)	20 (4.6)	49 (11.3)	-	-	-	49 (11.3)
Dizziness	22 (5.1)	23 (5.3)	43 (9.9)	1 (0.2)	1 (0.2)	2 (0.5)	44 (10.2)
Weight increased	12 (2.8)	25 (5.8)	37 (8.5)	-	3 (0.7)	3 (0.7)	40 (9.2)
Thrombocytosis	27 (6.2)	7 (1.6)	34 (7.9)	1 (0.2)	-	1 (0.2)	35 (8.1)
Haemoglobin decreased	15 (3.5)	16 (3.7)	29 (6.7)	-	4 (0.9)	4 (0.9)	32 (7.4)
Nasopharyngitis	27 (6.2)	1 (0.2)	28 (6.5)	-	-	-	28 (6.5)
Pruritus	22 (5.1)	5 (1.2)	27 (6.2)	-	-	-	27 (6.2)
Hypertension	24 (5.5)	3 (0.7)	27 (6.2)	-	-	-	27 (6.2)
Thrombocytopenia	9 (2.1)	16 (3.7)	24 (5.5)	-	1 (0.2)	1 (0.2)	25 (5.8)
Herpes zoster	13 (3.0)	10 (2.3)	23 (5.3)	1 (0.2)	1 (0.2)	2 (0.5)	25 (5.8)
Arthralgia	21 (4.8)	2 (0.5)	23 (5.3)	1 (0.2)	-	1 (0.2)	24 (5.5)
Epistaxis	15 (3.5)	5 (1.2)	20 (4.6)	4 (0.9)	-	4 (0.9)	24 (5.5)
Fall	13 (3.0)	-	13 (3.0)	11 (2.5)	-	11 (2.5)	22 (5.1)

*ADR* adverse drug reaction, *AE* adverse event, *AS* analysis set, *JAK* Janus Kinase, *nr* not related, *ns* non-serious, *SADR* serious adverse drug reaction, *SAE* serious adverse event

*AEs *without information on relation to study drug or seriousness were considered as related or serious, respectively

Overall, in 15 patients (3.5%) basal cell carcinoma was reported as AE, out of these, 0.2% (*n* = 1) were serious and drug related. Squamous cell carcinoma of skin occurred in 12 patients (2.8%), none of these AEs was reported as serious and drug related.

Within the MedDRA system organ class “infections and infestations” 140 patients (32.3%) had at least one AE. Here, the most common AEs (≥ 2% of the patients) were nasopharyngitis (6.5%, n = 28), herpes zoster (5.8%, n = 25), urinary tract infection (4.2%, n = 18), pneumonia (3.5%, n = 15), bronchitis (2.5%, n = 11), cystitis and infection (each 2.1%, 9 patients).

In 0.7% (n = 3) of the patients, acute myeloid leukemia was reported as AE and in 0.2% (n = 1) transformation to acute myeloid leukemia. In 0.5% of the patients (n = 2) each, myelofibrosis or myeloproliferative neoplasm were documented as AEs.

Cases of staphylococcal sepsis (0.7%, n = 3, outcome for all recovered), sepsis (0.2%, n = 1, outcome was recovered), septic shock (0.2%, n = 1, outcome was fatal), and urosepsis (0.2%, n = 1, outcome was recovered) were rare.

Forty-five non-related SAEs with fatal outcome (experienced by 36 patients, some patients experienced more than one SAE) occurred. Four SAEs causally related to treatment with JAK inhibitor and with fatal outcome (experienced by 4 patients) were reported: for all of them the causality regarding treatment with JAK inhibitor was not reported by the physicians. Due to this missing information this death cases were assessed with the worst case relationship (e.g., related) by the sponsor, although a non-causal relationship regarding treatment with JAK inhibitor was also possible. The 4 documented terms were: “death” (thrice) and “chronic lymphocytic leukaemia”.

## Discussion

The aim of this NIS was to obtain data from standard clinical routine on ruxolitinib therapy of PV in a broad patient population. In order to assess the immediate treatment effect, patients without prior treatment with ruxolitinib (naïve patients) were included in this NIS; at the same time, pretreated patients were documented in order to better investigate the long-term effectiveness of ruxolitinib therapy. Furthermore, patients were stratified by risk class, whereby high risk was defined as older than or equal to 60 years and/or history of thromboembolic events.

Sustained HCT < 45% is of major importance in PV patients in order to reduce thrombotic risk. Other disease-induced factors, such as leukocytosis, thrombocytosis and an enlarged spleen also play an important role in the disease management of the patients [10, 20].

This study demonstrated rapid and sustained effectiveness of ruxolitinib in the forementioned manifestations of PV in the majority of patients under routine use. Mean HCT levels rapidly decreased and, over time, were lower in JAK inhibitor naive patients compared with pretreated patients and in high-risk patients compared with low-risk patients. HCT levels were effectively reduced in most patients without the need for phlebotomy interventions. The incidence of thromboembolic events was low, with 3.5% of patients (*n* = 15) experiencing at least one such event. Mean leukocyte counts over time were similar in JAK inhibitor naive and pretreated patients and were lower in low risk than high-risk patients. Mean thrombocyte counts over time were similar in JAK inhibitor naive and pretreated patients and were lower in high-risk than low-risk patients. Most of the evaluable patients achieved a decrease from Baseline in spleen length. Symptom burden improved substantially in JAK-inhibitor naïve patients, JAK-inhibitor pretreated patients who benefited from prior therapy maintained their modified MPN-10 scores. Decreases in symptom burden were greater in patients without high risk compared to patients with high risk, for the latter symptom burden remained stable over time.

Inferentially, ruxolitinib led to benefits in hematocrit, leukocyte, and thrombocyte levels, as well as spleen length, regardless of prior JAK-inhibitor treatment or risk status at Baseline. Generally, JAK-inhibitor naïve patients had greater improvements than JAK-inhibitor pretreated patients. However, JAK-inhibitor pretreated patients continued to show benefit.

Ruxolitinib demonstrated a safety profile consistent with what had been previously reported. It was continuously well tolerated, with the majority of the patients (82.4%, *n* = 357) having no interruption of ruxolitinib therapy. Out of the total number of AEs (*n* = 1970), 53 SAEs were assessed as related (2.7%) to treatment with JAK inhibitor. However, because the patients received HU and other treatment options prior to this study the causal relationship between ruxolitinib and the AEs is difficult to determine [21]. Anemia was the most frequent adverse event, as expected, given the mechanism of action of ruxolitinib. Here, cases of anemia were mostly non-serious and of mild to moderate intensity.

Due to its mechanism of action ruxolitinib has immunosuppressive effects, as a result an increased rate of infection was observed in clinical studies and in practical application. In the pivotal RESPONSE trial, which included HU resistant or intolerant PV patients with a spleen volume of > 450 cm^3^, the overall infection rate in the ruxolitinib group was 41.8% and 36.9% in the best available therapy group [22]. In our study, the overall infection rate was 32.3%. The most frequent infections were nasopharyngitis (6.5%, *n* = 28) and herpes zoster (5.8%, *n* = 25). Most of the herpes zoster infections were non-serious, of mild/moderate intensity, and were resolved. Only one patient each experienced a serious and drug-related herpes zoster infection/reactivation. Of note, 11 patients had herpes zoster documented as concomitant disease at baseline. Sadjadian et al. recommends a careful assessment of the risk of infection for PV patients prior to the start of treatment with ruxolitinib [22].

As clinical experience grows, so does the amount of information on the occurrence of non-melanoma skin cancers under treatment with ruxolitinib. This is of particular interest as there is concern about the potential carcinogenic effect of ruxolitinib. Here, cases of serious and drug-related squamous cell carcinoma or basal cell carcinoma were very rare (0.2% each). Since HU treatment might also be responsible for development of precancerous lesions and non-melanoma skin cancer, the previous HU therapy might be a contributing factor for non-melanoma skin cancer [23].

Only very few patients (less than 1% each) experienced acute myeloid leukemia, transformation to acute myeloid leukemia, or myelofibrosis.

The rate of patients with a weight increase (9.2%), hypertension (6.2%), or hypercholesterolaemia (0.5%) was also comparable with previous clinical studies. The control of cardiovascular risk factors is crucial in patients with PV, and patients should be monitored and treated according to clinical guidelines [18].

Overall, incidence of AEs was slightly lower in JAK-inhibitor pretreated patients compared JAK-inhibitor naïve patients. Possibly, the management of AEs was better in JAK-inhibitor pretreated patients compared to JAK-inhibitor naïve patients due to the longer duration of treatment and thus successful treatment of those AEs by the physicians.

Overall, ruxolitinib therapy was well tolerated and no new safety concerns were observed. The safety and tolerability results are crucial in this context, due to the long-term nature of the therapy required to control PV.

The study was performed under standard clinical practice conditions in Germany and allowed the enrollment of a heterogeneous patient population with regard to demographic and disease characteristics. Patients including a wide age range were enrolled. The observational design of the study allowed to collect real-world data, without influencing the physicians’ treatment decisions.

In 2024, Theocharides et al. published the results of a phase IV, European, real-world, observational study which assessed the effectiveness and safety of ruxolitinib in PV patients who were resistant/intolerant to HU, with a 24-month follow-up [24]. 350 patients were enrolled. In addition to the prospective patients who were going to be prescribed ruxolitinib, retrospective patients who had already initiated treatment ≤ 6 months before the date of the informed consent signature were also included. Baseline characteristics were largely comparable to our study, however, PaVe patients were slightly older than the patients in the European study (83% ≥ 60 years versus 70% ≥ 60 years) and started from a lower mean HCT level (40.5% versus 45.2%). Both studies corroborate real-world effectiveness of ruxolitinib in HU-resistant/intolerant PV: rapid, durable HCT control, fewer phlebotomies, symptom and spleen improvements. The European study quantifies strict HCT control (68.2% at 24 months), while PaVe demonstrates sustained sub-45% HCT through 36 months, particularly in JAK inhibitor-naïve patients. The higher SAE rate in PaVe (37.0% versus 19.1% in the European study) likely reflects older age and longer follow-up in the PaVe study.

However, this study was a NIS with the limitations associated with all observational studies, including selection bias due to lack of randomization, confounding bias with respect to heterogeneity of the observed population, and outcome bias with respect to the completeness and consistency of data. As the treating physician decides on the prescription of the respective medication and inclusion of the patient in the NIS, this may influence the patients’ decisions and course of treatment, thereby introducing a bias. Patients who discontinue the documentation during the observation period may create an outcome bias. Missing values and decreasing numbers of available patients and observations over time may hamper the interpretability of results.

In conclusion, this study confirmed data from clinical studies (RESPONSE, RESPONSE 2, and MAJIC-PV [18–21, 25]) that ruxolitinib led to benefits in HCT, leukocyte, and thrombocyte levels, as well as spleen length regardless of prior JAK inhibitor treatment or risk status at Baseline in patients with PV under real-world conditions. No new safety signals were detected. Taken together, findings from this study in a real-world setting show that ruxolitinib is an effective and safe second-line treatment in PV patients.

## Data Availability

The datasets generated and/or analysed during the study are not publicly available but are available from the corresponding author on reasonable request and with permission of Novartis.
